# CAN THE PERCUTANEOUS CHEVRON AND AKIN (PECA) TECHNIQUE CORRECT THE PRONATION OF THE FIRST METATARSAL IN HALLUX VALGUS?

**DOI:** 10.1590/1413-785220233102e265206

**Published:** 2023-06-09

**Authors:** GABRIEL FERRAZ FERREIRA, MAURO CESAR MATTOS E DINATO, TATIANA FERREIRA DOS SANTOS, PAULO MIZIARA, MIGUEL VIANA PEREIRA

**Affiliations:** 1. Prevent Senior, Foot and Ankle Surgery Group, Orthopaedics and Traumatology Unit, São Paulo, Brazil.; 2. Prevent Senior, Head of Foot and Ankle Surgery Group, Orthopaedics and Traumatology Unit, São Paulo, Brazil.; 3. Instituto Vita, São Paulo, Brazil.

**Keywords:** Hallux valgus, Minimally invasive surgical procedures, Pronation, Hallux valgus, Procedimentos cirúrgicos minimamente invasivos, Pronação

## Abstract

**Objective:**

Pronation of the first metatarsal in hallux valgus has recently been discussed among foot and ankle surgeons. This study aimed to evaluate the potential radiographic correction of moderate and severe hallux valgus using the percutaneous Chevron and Akin (PECA) technique.

**Methods:**

We evaluated 45 feet in 38 patients (mean age 65.3 years old [36 - 83]; 4 men; 34 women; 7 bilateral) who underwent surgical correction using the PECA technique. The radiographic images evaluated were anteroposterior radiographs obtained pre- and postoperatively at least 6 months after surgery, including the metatarsophalangeal angle, the intermetatarsal angle, pronation of the first metatarsal, displacement of the distal fragment, medial sesamoid position and bone union.

**Results:**

All parameters evaluated showed significant postoperative improvement, including correction of pronation of the first metatarsal (p < .05) and position of the sesamoid (p < .05). There was a union of osteotomies in all feet. No complications were observed, such as screw loosening or necrosis of the first metatarsal head.

**Conclusion:**

The PECA technique can correct pronation of the first metatarsal in moderate and severe hallux valgus, and other deformity-associated parameters. Level of Evidence IV; Case Series.

## RESUMO

**Objetivo:**

A pronação do primeiro metatarso no hálux valgo tem sido um tema de discussão recente entre os cirurgiões de pé e tornozelo. O objetivo deste estudo foi avaliar o potencial de correção radiográfica do hálux valgo moderado e grave utilizando a técnica percutânea de Chevron e Akin (PECA).

**Métodos:**

Avaliamos 45 pés em 38 pacientes (média de idade 65,3 anos [36 - 83]; 4 homens; 34 mulheres; 7 bilaterais) submetidos à correção cirúrgica pela técnica PECA. As imagens radiográficas avaliadas foram radiografias anteroposteriores obtidas no pré e pós-operatório com no mínimo 6 meses após a cirurgia, incluindo ângulo metatarsofalângico, ângulo intermetatarsal, pronação do primeiro metatarso, deslocamento do fragmento distal, posição do sesamoide medial e união óssea.

**Resultados:**

Todos os parâmetros avaliados apresentaram melhora significativa no pós-operatório, incluindo correção da pronação do primeiro metatarso (p < 0,05) e posição do sesamoide (p < 0,05). Houve união de osteotomias em todos os pés. Não foram observadas complicações, como soltura do parafuso ou necrose da cabeça do primeiro metatarso.

**Conclusão:**

A técnica PECA pode corrigir a pronação do primeiro metatarso no hálux valgo moderado e grave, bem como outros parâmetros associados à deformidade. Nível de Evidência IV; Série De Casos.

## INTRODUCTION

Hallux valgus is a complex disorder of the foot and can be considered a deformity in three planes: frontal, transverse and sagittal.^1^ Coronal deformity, which is characterized by pronation of the first metatarsal, is present in up to 87% of individuals with hallux valgus.^2^

If not corrected in surgical treatment of hallux valgus, pronation of the first metatarsal is associated with an increased chance of recurrence.^3^ Correction of rotational deformity through the open technique using distal and proximal osteotomies and cuneometatarsal arthrodesis has been described.^4 - 8^

The percutaneous chevron and Akin (PECA) technique, which was developed by Vernois and Redfern, is among the third generation of percutaneous surgeries for hallux valgus correction and uses stable internal fixation with screws.^9^

Studies comparing PECA with open techniques have consistently demonstrated that the percutaneous technique obtains clinical and radiographic results similar to those of open techniques^10 , 11^ but with reduced scar and pain in the postoperative period.^12 - 14^ However, whether the PECA technique acts in the coronal plane and promotes correction of the pronation of the first metatarsal has not been evaluated.

The objective of this study was to evaluate the correction of hallux valgus associated deformities in both the transverse and coronal planes in a series of patients who underwent hallux valgus correction using the PECA technique.

## METHODS

Radiographic analysis was performed of a series of consecutive cases of patients diagnosed with hallux valgus undergoing surgical treatment using the PECA technique between August 2018 and December 2019. The surgeries were performed by the same team, in a single center, supervised by the senior author (MVPF), experienced in minimally invasive surgery. The study was approved by the local ethics committee (Plataforma Brasil Protocol CAAE: 32878720.0.0000.5474) and followed the Declaration of Helsinki and the Guidelines for Good Clinical Practice.

The total number of patients undergoing surgical treatment was 38, of whom 7 were bilateral, totaling 45 feet. The mean age was 65.3 years with a standard deviation of 10.7, and the age range was 36 to 83 years. The left foot was the most operated (53.3%), and most patients were female (90.5%).

The inclusion criteria were as follows: (1) patients with clinical and radiographic diagnosis of moderate and severe hallux valgus^15^ (metatarsophalangeal angle greater than 20 degrees and intermetatarsal angle greater than 11 degrees) and (2) symptomatic patients without improvement with at least 6 months of conservative treatment. Patients with sequelae or previous surgeries of the foot and ankle, incomplete data or inadequate radiographic images, Charcot arthropathies, active infection or decompensated diabetes mellitus were excluded from the present study.

The distal metatarsal metaphyseal osteotomy (DMMO) procedure for the percutaneous treatment of metatarsalgia, the method for which has been described in several published studies,^16 - 20^ was performed in combination with hallux valgus correction using the PECA technique in 19 feet (43%).

All patients were operated on by the same team and supervised by a surgeon experienced in minimally invasive foot and ankle procedures.

### Surgical technique and postoperative care

The surgical technique used in this study was a modification of the technique originally described by Vernois and Redfern.^9^ We did not fix any Akin osteotomy, which was maintained only with bandage.

The patients underwent the procedure in the supine position on the operating table, and no tourniquet was used on the limb. We identified the site for osteotomy according to clinical and fluoroscopic markings.

An approximately three-millimeter incision was made using a specific scalpel blade in the region of the proximal curve of the medial eminence of the first metatarsal. A space was created by placing a periosteal elevator for percutaneous surgery to facilitate the introduction of the burr in the region proximal to the joint capsule.

Chevron osteotomy was then performed with a 2 x 20 mm *Shannon* -type burr positioned in the neck of the first metatarsal. The dorsal portion of the osteotomy was straight and perpendicular to the dorsal cortex. The plantar portion was performed at an angle to prevent dorsal displacement of the head.

Next, a periosteal elevator was inserted into the intramedullary canal through the same incision as described by Lai et al.^12^ by moving the distal fragment as far as possible to the lateral region.

The control of the position of the distal fragment was determined by the image intensifier. At no time was there an attempt to supine the distal fragment; only the distal fragment was transferred, and the alignment was maintained in the sagittal plane. To facilitate displacement, the surgeon pulled and performed slight varization of the hallux.

Next, a 2.0-mm Kirchner wire was used to create the screw path approximately four to 5 centimeters proximal to the first incision, from the base of the first metatarsal to the central region of the head, always passing through the two proximal cortical sites. Then, the wire was percutaneously removed, the guidewire of the screws was inserted, and the osteotomy was fixed with two cannulated screws.

Lateral release of the soft tissues was performed percutaneously and only in cases where the surgeon judged that there was a contracture of the lateral structures perpetuating the valgus deformity. Lateral release was always performed after fixation of the head with a specific scalpel blade lateral to the extensor hallucis longus tendon.

After release, in cases where some degree of valgus persisted, Akin osteotomy was performed. The Akin procedure required a new incision, always distal to the chevron osteotomy incision, performed with the same burr and guided by the image intensifier. No osteotomy was fixed.

Finally, the surgical area was cleaned with saline solution, and the incisions were sutured with 4-0 nylon. The postoperative dressing included dry gauze, gauze strips to stabilize and position the toes and finally a crepe and elastic bandage. The patients were encouraged to perform immediate weight bearing using specific sandals with rigid soles. The dressing was changed weekly by the medical team for 3 weeks, and then the patient was instructed to change it at home for up to 4 weeks, depending on other procedures performed in combination with hallux valgus correction.

No antithrombotic drug prophylaxis was prescribed. All patients were discharged on the same day and returned to the outpatient clinic within one week.

### Radiographic evaluation

Radiographic images were taken by the same group of technicians with patients standing with full body weight and feet in the plantigrade position. The anteroposterior (AP) view was obtained, and radiographs considered inadequate were excluded from this study.

The angles considered in our evaluation were the metatarsophalangeal angle, better known as the hallux valgus angle (HVA), and the intermetatarsal angle (IMA). These angles were measured in the preoperative radiograph and in the last evaluation performed. We also evaluated the position of the sesamoid as described by Hardy and Clapham^21^ on a scale ranging from one to seven.

**Figure 1:**
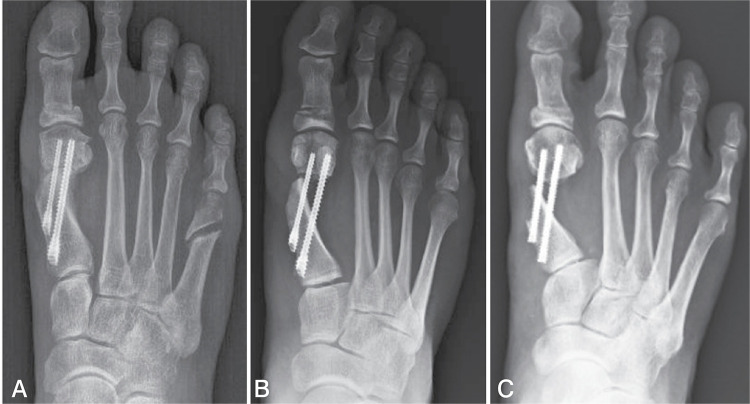
A) Displacement of the distal fragment less than or equal to 50%. B) Displacement of the distal fragment between and 50% and 100%. C) Displacement of the distal fragment greater than or equal to 100%.

The displacement of the distal fragment after osteotomy was divided into three different groups: less than or equal to 50% displacement (group I), greater than 50% and less than 100% (group II) and greater than or equal to 100% (group III), as shown in the examples in Figure 1 .

To better determine the degree of displacement, the first radiographic image following the surgical procedure was used to avoid images with calluses that could influence the result.

**Figure 2:**
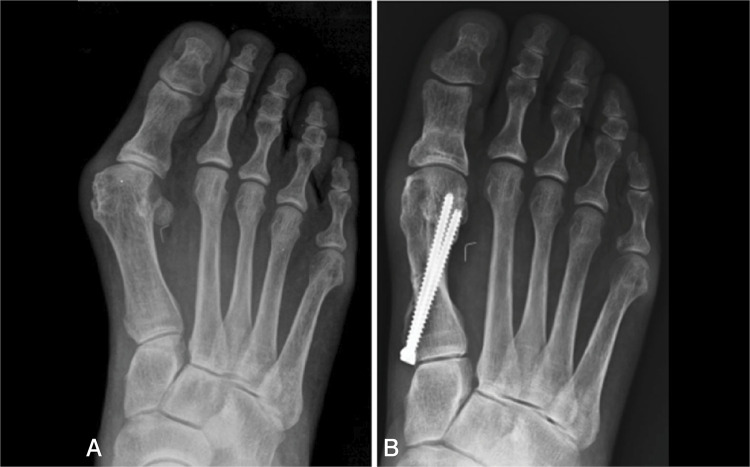
A) Preoperative image classified as pronation type R (round). B) Postoperative image classified as pronation type A (angular).

The stages of pronation were measured using AP radiographs with weight bearing considering the lateral shape of the first metatarsal head. ( Figure 2 )

We chose to use the classification of the first metatarsal pronation in the hallux valgus described by Okuda et al.,^3^ which was divided into type A (angular), type R (round) and type I (intermediate). As demonstrated by Yamagushi,^22^ the presence of a rounded contour in the lateral region of the head of the first metatarsal in the AP view is evidence of pronation because this contour is the silhouette of the lateral condyle.

The greater the pronation is, the more visible the contour of the condyle, resulting in a more rounded appearance of the lateral aspect of the head. Type A corresponds to the absence of pronation or mild pronation up to 20 degrees. Type I corresponds to moderate pronation between 20 and 30 degrees, and type R corresponds to pronation of 30 degrees or more.^23^

Three authors (P.M., T.F.S. and M.C.D.) performed the pre and postoperative classification independently. The result was recorded in a form that only the author (G.F.F.) had access to. In cases where there was no consensus, the senior author (M.V.P.F.) decided the classification.

Image analysis was performed digitally using Centricity® Universal Viewer Zero Footprint software (GE Healthcare, Barrington, IL, USA).

### Statistical data analysis

Continuous variables were tested for a normal distribution using the Shapiro-Wilk test.^24^ The Wilcoxon signed rank test^25^ and paired Student’s t test^26^ were used for comparisons of nonnormally and normally distributed data, respectively.

Comparisons between unpaired continuous variables were performed using Student’s t-test^26^ for parametric variables and the Mann-Whitney U test for nonparametric variables.

Categorical variables were measured using their proportion, and the chi-square test was performed.^27^ The Pearson correlation test was used to measure the degree of correlation between two numerical and parametric variables.

All statistical evaluations were performed using the software R,^28^ specifically using the *Stats* package, both open source. A value of *p* ≤ .05 was adopted as the statistical level of evidence.

## RESULTS

The mean postoperative follow-up time was 13.1 months with a standard deviation of 4.9 months; the postoperative follow-up time ranged from 6 to 22 months. There was no loss of follow-up of any patient. Most of the deformities were considered moderate and represented 51.1% of the sample; severe cases represented the remainder. Lateral soft tissue release was required in only 33.3% of cases.

Moderate or severe pronation of the first metatarsal was found in the preoperative radiographic images in 32 feet (71.1%), of which 20 were type I and 12 were type R. After the surgical procedure, the pronation was corrected (type A) in 14 feet (43.7%) feet, partially corrected (from type R to type I) in eight feet (25.0%) and unchanged in 10 feet (31.3%).

Type A pronation was evidenced in the preoperative radiographs in 13 feet, which represented 28.9% of the total sample. Of these, 11 maintained the same pronation, and two increased the pronation for type I after surgical correction.

The radiographic evaluation showed a statistically significant improvement in HVA (p < .001), IMA (p < .001) and pronation of the first metatarsal (p < .05), as shown in Table 1 .

**Table 1:** Pre and postoperative radiographic results of hallux valgus measurements.

Outcome	Preoperative	Postoperative	Statistical analysis
HVA, mean and SD	40.1 ± 6.5	8.8 ± 7.6	p < .001
IMA, mean and SD	16.1 ± 3.2	5.6 ± 4.0	p < .001
Pronation of the first metatarsal	Type A = 13 (28.9%)	Type A = 25 (55.6%)	p < .05
	Type I = 20 (44.4%)	Type I = 20 (44.4%)	
	Type R = 12 (26.7%)	Type R = 0 (0%)	

Abbreviations: HVA: hallux valgus angle; IMA: intermetatarsal angle; SD: standard deviation;

The final pronation of the first metatarsal had a mean HVA of 6.84 degrees in type A and 11.25 degrees in type I, with no significant difference (p = .057). Similarly, there was no significant difference in IMA (p = .89), with a mean IMA of 5.72 degrees for type A and 5.45 degrees for type I.

The displacement distribution of the distal fragment of the first metatarsal in the osteotomy was 35.5% group I, 37.8% group II and 26.7% group III.

**Figure 3:**
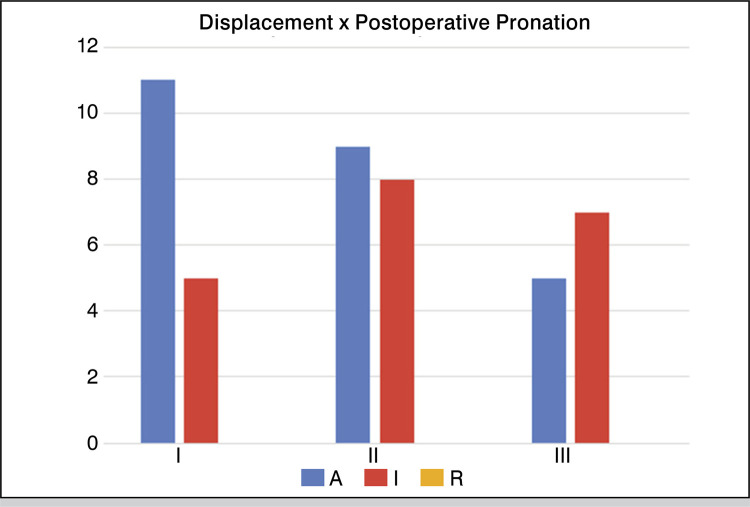
Relationship between displacement and postoperative pronation of the first metatarsal.

No relationship was found between the postoperative stages of pronation and the displacement groups obtained during the surgical procedure (p = .34), and the frequency of each group is shown in Figure 3 .

A statistically significant variation in the sesamoid position was observed after surgical correction (p <.05). The distribution of classifications between the groups is shown in Table 2 .

**Table 2:** Position of the sesamoid on AP radiography before and after surgery.

Grade	Preoperative	Postoperative
I	0	4 (8.9%)
II	0	12 (26.7%)
III	0	15 (33.4%)
IV	0	7 (15.5%)
V	3 (6.7%)	6 (13.3%)
VI	14 (31.1%)	1 (2.2%)
VII	28 (62.2%)	0
Normal position (Grade I to IV)	0	38 (84.4%)
Incongruent Position (Grade V to VII)	45 (100%)	7 (15.6%)

Statistical analysis with the chi-square test (p < .05)

The correlation between HVA before and after surgery showed a Pearson correlation coefficient of -0.01, without statistical significance (p = .94).

### Complications

All osteotomies resulted in a bone union. We did not observe any loosening or breakage of the screw. No patient with necrosis of the first metatarsal head was identified. No patient underwent any additional surgical procedure.

## DISCUSSION

The patients included in this study had mean preoperative HVA and IMA of 40.1 and 16.1 degrees, respectively. Thus, the cases in this series are, on average, more severe cases than those previously described in reports using the same technique. In the other series in which the PECA technique was used, the preoperative HVA ranged between 25 and 31.4 degrees, and the IMA ranged between 11.7 and 15.6 degrees.^10 - 14 , 29^

Despite having patients with more pronounced deformities, the radiographic results at the end of the follow-up were similar to those of the other series. Our patients had mean final HVA and IMA of 8.8 degrees and 5.6 degrees, respectively, while in previous studies, mean final HVA and IMA varied between 8.5 and 10.9 degrees and between 5 and 10.2 degrees, respectively.

In our series, 24 feet had preoperative HVA between 40 and 50 degrees, and three feet had HVA above 50 degrees. There was no correlation between the preoperative HVA values and the values achieved after the surgical procedure, which indicates that the PECA technique can potentially be used in both severe and moderate cases.

Okuda et al.^3^ demonstrated that the presence of pronation of the first ray and incomplete reduction of the sesamoid in the postoperative period are risk factors for hallux valgus recurrence. Pronation is characterized by a positive round sign, where the lateral cortical of the first metatarsal head has a rounded shape and causes dorsolateral deviation of the sesamoid. In the PECA technique, osteotomy is performed on the metatarsal neck, and the head is pushed to the side, while the hallux is pulled and subjected to slight varization.

We believe that this maneuver naturally corrects the pronation of the toe and by ligamentotaxis corrects the position of the head. All patients who had a round sign in the preoperative period did not present this sign in the postoperative period. In addition, the percentage of patients who were classified as type A in the preoperative period, indicating mild or absent pronation, increased from 29 to 55% after surgery (p < .05).

We did not identify any other study in which the PECA technique was used and which postoperative rotation was evaluated. There are few described techniques that can correct pronation of the first metatarsal. Most procedures were developed for lateral displacement and varus correction, such as the open chevron and Scarf procedures, and are unable to perform rotation correction.^23^

Procedures that allow correction of head rotation include rotational biplanar chevron osteotomy,^4^ proximal oblique sliding closing wedge osteotomy,^6^ proximal supination osteotomy,^7^ proximal rotational metatarsal osteotomy^8^ and arthrodeses, such as the Lapidus procedure^30^ and metatarsophalangeal arthrodesis.

Yasuda and colleagues obtained a significant decrease in pronation by using a crescentic osteotomy at the base of the first metatarsal.^7^ In the preoperative period, 83% of patients had a positive round sign, compared to 20% in the postoperative period.

There was no difference between the correction of pronation and the degree of displacement of the head, which indicates that in this sample, it was not necessary to translate more than 100% of the head in relation to the metatarsal for the correction of rotation. The burr, with a thickness of 2 mm, makes a cut that allows some degree of rotation, even when there is contact between the head and the diaphysis.

In addition, we performed an oblique plantar portion of the osteotomy to avoid dorsal displacement of the distal fragment. However, this type of osteotomy could limit the rotation of the head to some degree in cases where the displacement is less than 100%. In theory, straight osteotomies would facilitate rotation.

Shibuya et al. conducted a retrospective cohort study to identify the predictive factors of hallux valgus recurrence.^31^ The only associated factor was the position of the medial sesamoid in the postoperative period, with rates of 50 and 60% relapse when the position was greater than four or five, respectively.

The evaluation of the sesamoid position in our series showed that there was a significant correction. The percentage of feet with the sesamoid in the normal position (less than or equal to four) ranged from 0 in the preoperative period to 84% in the postoperative period (p < .05). As there is a relationship between pronation and sesamoid position,^32^ these numbers reinforce the hypothesis that there was indeed pronation correction in most patients.

As we only analyzed radiographic results, the evaluation of complications was limited. In 26% of patients, head displacement was performed above 100% of the diaphysis width, resulting in absence or minimal contact between the fragments and raising concerns about non union. However, union occurred in all patients. The fact that the osteotomy was percutaneous, with minimal aggression to the soft tissue envelope, most likely contributed to these results.

The study has some limitations. The first is the evaluation of rotation by means of AP radiography of the foot. Studies are still needed to demonstrate whether this method has intra and interobserver reliability and is comparable to computed tomography, which probably the most accurate method for performing this evaluation. Another important limitation is the relatively short follow-up, which was as little as 6 months in some cases and may have overestimated the potential for correction using this technique if there is recurrence of rotation over time. Finally, the mean age of the sample was above that of the general population, which limits the analysis for other age groups.

## CONCLUSION

The percutaneous chevron and Akin technique can correct pronation of the first metatarsal in moderate and severe hallux valgus, as well as other parameters associated with this deformity.
